# Properties and Crystal Structure of Methylenetetrahydrofolate Reductase from *Thermus thermophilus* HB8

**DOI:** 10.1371/journal.pone.0023716

**Published:** 2011-08-15

**Authors:** Sayaka Igari, Akashi Ohtaki, Yasuaki Yamanaka, Yuichi Sato, Masafumi Yohda, Masafumi Odaka, Keiichi Noguchi, Kazuhiro Yamada

**Affiliations:** Department of Biotechnology and Life Science, Tokyo University of Agriculture and Technology, Koganei, Tokyo, Japan; University of Minnesota, United States of America

## Abstract

**Background:**

Methylenetetrahydrofolate reductase (MTHFR) is one of the enzymes involved in homocysteine metabolism. Despite considerable genetic and clinical attention, the reaction mechanism and regulation of this enzyme are not fully understood because of difficult production and poor stability. While recombinant enzymes from thermophilic organisms are often stable and easy to prepare, properties of thermostable MTHFRs have not yet been reported.

**Methodology/Principal Findings:**

MTHFR from *Thermus thermophilus* HB8, a homologue of *Escherichia coli* MetF, has been expressed in *E. coli* and purified. The purified MTHFR was chiefly obtained as a heterodimer of apo- and holo-subunits, that is, one flavin adenine dinucleotide (FAD) prosthetic group bound per dimer. The crystal structure of the holo-subunit was quite similar to the *β_8_α_8_* barrel of *E. coli* MTHFR, while that of the apo-subunit was a previously unobserved closed form. In addition, the intersubunit interface of the dimer in the crystals was different from any of the subunit interfaces of the tetramer of *E. coli* MTHFR. Free FAD could be incorporated into the apo-subunit of the purified Thermus enzyme after purification, forming a homodimer of holo-subunits. Comparison of the crystal structures of the heterodimer and the homodimer revealed different intersubunit interfaces, indicating a large conformational change upon FAD binding. Most of the biochemical properties of the heterodimer and the homodimer were the same, except that the homodimer showed ≈50% activity per FAD-bound subunit in folate-dependent reactions.

**Conclusions/Significance:**

The different intersubunit interfaces and rearrangement of subunits of Thermus MTHFR may be related to human enzyme properties, such as the allosteric regulation by *S*-adenosylmethionine and the enhanced instability of the Ala222Val mutant upon loss of FAD. Whereas *E. coli* MTHFR was the only structural model for human MTHFR to date, our findings suggest that Thermus MTHFR will be another useful model for this important enzyme.

## Introduction

The folate cofactor is a carrier of a C1 unit, which is a functional group consisting of a single carbon in various states of reduction (CHO-, CH = , CH_2_-, CH_3_-). For living cells, especially growing cells, synthesis of DNA, RNA, and protein is essential. Because the C1 unit on folate can be utilized to produce purine bases, thymidylate, and methionine, folate and methionine metabolism is important to provide materials for these biosyntheses. Methylenetetrahydrofolate reductase (MTHFR) is one of the key enzymes for production of methionine, which is not only an important amino acid precursor for protein synthesis, but also the precursor of *S*-adenosylmethionine (AdoMet), which serves as a major methyl donor and a substrate for polyamine synthesis. In addition, AdoMet acts as a strong allosteric inhibitor for mammalian MTHFR 1]. This classic negative feedback loop regulates methionine biosynthesis.

Hyperhomocysteinemia, an elevated level of homocysteine concentration in blood, is an independent risk factor for cardiovascular disease 2]. MTHFR is one of the enzymes that plays a crucial role in homocysteine metabolism. Although homocysteine is not a substrate for MTHFR, methyltetrahydrofolate (CH_3_-H_4_folate), the enzyme product, and homocysteine are used by cobalamin-dependent methionine synthase to produce methionine. Hence, dysfunction of MTHFR can lead to hyperhomocysteinemia. Many clinical and epidemiological studies have described the relation between MTHFR gene mutations, particularly a 677C→T common polymorphism 3], and human disease.

**Figure 1:**
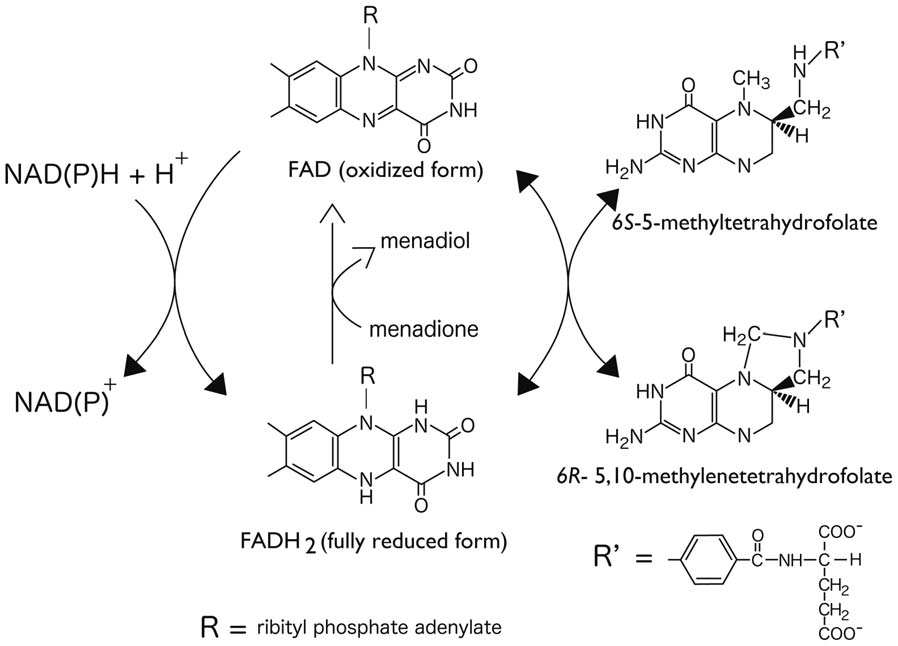
Reactions catalyzed by MTHFR. MTHFR non-covalently binds FAD as an essential cofactor. NAD(P)H and CH_2_-H_4_folate are physiological substrates. NAD(P)H reduces FAD, then the reduced FAD reduces CH_2_-H_4_folate. CH_3_-H_4_folate can reduce the oxidized FAD. The reduced FAD can be oxidized by menadione as an electron acceptor, which is routinely used for *in vitro* assays.

MTHFR requires flavin adenine dinucleotide (FAD) as a non-covalently bound cofactor for catalytic function 4]. As shown in Figure 1, MTHFR uses NAD(P)H to reduce FAD; subsequently the reduced FAD reduces methylenetetrahydrofolate (CH_2_-H_4_folate). Electron transfer between CH_3_-H_4_folate and the oxidized form of FAD is reversible, allowing assay of the enzyme by monitoring the oxidation of CH_3_-H_4_folate in the presence of an electron acceptor like menadione. These biochemical properties have been extensively analyzed using porcine MTHFR. Eukaryotic MTHFR is a homodimer and each subunit (70∼77 kDa) comprises a catalytic domain and a regulatory domain. *Escherichia coli* MTHFR (MetF protein), which consists of four smaller catalytic subunits (≈33 kDa), has been also used to investigate catalytic functions of MTHFR. In addition, effects of the 677C→T mutation of human MTHFR gene on the properties of the enzyme were revealed using an *E. coli* MTHFR mutant 5], indicating that bacterial MTHFR would be a useful model to study the human enzyme's properties. These studies demonstrated that Ala222 to Val mutation, which is caused by the 677C→T gene mutation, leads to enhanced loss of the flavin cofactor and is accompanied by subunit dissociation and sensitivity to thermal denaturation. Although several catalytically important amino acid residues in the active site of MTHFR have been identified from the structural and biochemical studies using *E. coli* enzyme 6–8], details about the reaction mechanism are not yet fully understood. Moreover, it is still undetermined how the activity of mammalian MTHFR is regulated by AdoMet.

To date, properties of MTHFR from various species, including pig 4], yeast 9], plant 10], human 11,12], *E. coli* 13], *Peptostreptococcus productus* 14] and *Clostridium formicoacetium* 15], have been reported. These enzymes are mostly active at normal temperature (24∼37°C). Enzymes from thermophilic organisms often show a slow rate in their reactions at room temperature, which may enable detection of intermediates, so MTHFR from thermophilic organisms could serve as a model to study the reaction mechanism. So far, biochemical properties of MTHFR from thermophilic organisms have not been reported, while the crystal structure of MTHFR from *Thermus thermophilus* HB8 as a monomeric protein has already been deposited in the Protein Data Bank (PDB, http://www.pdb.org/accession number 1V93). Here, we report biochemical properties and unique crystal structures of MTHFR from *T. thermophilus* HB8, one of the model organisms in structural biology. We demonstrate that the Thermus MTHFR is a dimer, rather than a tetramer, so that its symmetry is perhaps more directly relevant to the dimeric mammalian MTHFR. Furthermore, we show that the subunit packing in the dimer is modulated by the FAD content of the dimer, and suggest that similar changes in subunit packing may occur during the allosteric transition of mammalian MTHFR induced by AdoMet. Such rearrangements in subunit packing might also be associated with flavin loss in the Ala222Val mutant, and thus might underlie the dramatic increase in thermal sensitivity in the mutant human MTHFR.

## Methods

### Reagents

(*6S*)-tetrahydrofolate (H_4_folate) was purchased from Merck Eprova AG (Schaffhausen, Switzerland). (*6RS*)-5-CH_3_-H_4_folate (sodium salt) was purchased from Sigma-Aldrich (St. Louis, MO). NADH, biochemical grade, was purchased from Wako Pure Chemical (Osaka, Japan). All other reagents were analytical grade and were used without further purification.

### Enzyme Expression and Purification

The gene for *T. thermophilus* HB8 MTHFR was obtained from the Riken *Thermus thermophilus* HB8 expression plasmid set 16]. The clone number TTHA0327, encoding the wildtype MTHFR (*E. coli* MetF homologue) cloned into a pET11 vector, was used for non-His-tagged protein expression. To produce the enzyme as a C-terminally hexa-His-tagged form, the wildtype MTHFR gene was amplified by polymerase chain reaction and cloned into a pET23 vector (Novagen, Merck KGaA, Darmstadt, Germany). The expression vector was designated as pET(tMR^wt^-H). DNA and deduced amino acid sequences of the vector are shown in Figure S1. *E. coli* BL21(DE3) was transformed using these expression vectors. Recombinant proteins were produced in LB Broth (Merck) containing ampicillin and 0.1 mM isopropyl-*β*-D-thiogalactopyranoside at 25°C.

Non-tagged and His-tagged enzymes were extracted into 50 mM potassium phosphate buffer, pH 7.2 (KPB) containing 0.2 M sodium chloride (NaCl) in the presence of 1 mM phenylmethylsulfonyl fluoride by sonication. The sonicate was centrifuged at 20,000 xg for 15 min at 4°C; then the supernatant was recovered. The supernatant was treated with incubation at 70°C for 20 min, then centrifugation, followed by filtration. The filtrate containing non-tagged wildtype enzyme was passed through a Toyopearl SuperQ-650M column (Tosoh, Tokyo, Japan). Because of the high pI of Thermus MTHFR, the target protein did not bind to the anion exchange column whereas most of other large molecules, such as other proteins and DNA, were adsorbed. Yellow-colored through fractions were pooled and dialyzed against 0.1 M KPB, containing 0.1 M NaCl and 1 mM EDTA, overnight at 4°C. The dialyzate was concentrated and stored. His-tagged wildtype enzyme was purified using a Ni-affinity column (Hi-Trap, Chelating HP, GE Healthcare Amersham Biosciences AB, Uppsala, Sweden). After heat treatment of the sonicate, the extract was loaded onto the Ni-column. The column was washed with 50 mM KPB, containing 0.2 M NaCl and 100 mM imidazole, then MTHFR was eluted with 50 mM KPB containing 0.2 M NaCl and 300 mM imidazole. The yellow eluate was collected. The pooled fraction was dialyzed and concentrated as in the preparation of the non-His tagged enzyme. Enzyme concentration was calculated from FAD concentration using the molar extinction coefficient (14.1×10^3^) at 450 nm for bacterial MTHFR 13] and expressed as [Et]. Thus, [Et] indicates the concentration of FAD-bound subunits. Enzyme preparations from this method are referred to as ‘as-purified’ MTHFR.

To prepare fully FAD-replete purified MTHFR, the as-purified MTHFR ([Et]  =  200∼300 µM) at a volume of ≈1 ml was incubated with an excess amount of FAD (≈5 mM) at 37°C for 1 hr. After incubation, the mixture was passed through a size exclusion column (EconoPac 10 (Bio-Rad Laboratories, Hercules, CA)) equilibrated with 0.1 M KPB, 0.1 M NaCl, 1 mM EDTA, then the protein fractions were pooled and concentrated. Enzymes treated with FAD were designated as ‘FAD-replete’ MTHFR.

### Enzyme Assay

As shown in Figure 1, there are three ways to measure MTHFR activity: NADH:CH_2_-H_4_folate oxidoreductase activity, a physiological assay; NADH:menadione oxidoreductase activity, the reductive half reaction using menadione as an electron acceptor; and CH_3_-H_4_folate:menadione oxidoreductase activity, a reverse direction assay using CH_3_-H_4_folate and menadione. Enzyme assays, except for the reverse direction assay, were performed according to previous reports 4] 13] with minor modifications.

The NADH:CH_2_-H_4_folate oxidoreductase assay was performed using a photometric assay employing a Jasco UV-VIS photometer equipped with a cell temperature controller (Jasco Corp., Tokyo, Japan). (*6R*)-5,10-CH_2_-H_4_folate was prepared by the non-enzymatic reaction of (*6S*)-H_4_folate and formaldehyde. (*6S*)-H_4_folate was dissolved in Argon(Ar)-substituted 50 mM KPB containing 0.5 M 2-mercaptethanol at a final concentration of 10 mM, then, formaldehyde (final concentration was 20 mM) was added. Excess formaldehyde is needed to drive the condensation of formaldehyde with H_4_folate to form CH_2_-H_4_folate. The mixture was incubated at least 30 min at room temperature. Assay buffer (50 mM KPB) in a cuvette was preincubated at 50°C. NADH, enzyme, and CH_2_-H_4_folate were added into the cuvette, then the reaction was monitored at 340 nm.

NADH:menadione oxidoreductase activity was measured using a Cary 50 UV-VIS spectrophotometer (Agilent Technologies, Inc. Santa Clara CA). The reaction mixture without menadione was prepared at room temperature. To start the reaction, menadione was added to the cuvette and then changes in absorption at 343 nm were recorded at room temperature. For routine assays, 100 µM NADH and 100 µL saturated menadione solution per ml of reaction mixture were used.

CH_3_-H_4_folate: menadione oxidoreductase activity was determined without using radioactive materials. The principle of the measurement was originally designed for the assay of methionine synthase activity 17]. H_4_folate, a product of methionine synthase, can be converted to methenyltetrahydrofolate (CH^+^ = H_4_folate) in the presence of formic acid under the acidic conditions. The UV absorbance of CH^+^ = H_4_folate has a maximum at 350 nm, which is unique to this derivative of H_4_folate. This property was adapted to measure CH_3_-H_4_folate: menadione oxidoreductase activity. There is an equilibrium between H_4_folate and CH_2_-H_4_folate, the product of the reaction. Because of the equilibrium, product CH_2_-H_4_folate can be captured as CH^+^ = H_4_folate in the presence of formic acid, following dissociation to form H_4_folate. Enzyme activity was calculated using the concentration of CH^+^ = H_4_folate produced as determined by absorbance at 350 nm. Assays were done as follows: diluted Thermus MTHFR in degassed- and Ar-substituted- 50 mM KPB was preincubated at 50°C and then menadione stock solution and enzyme were added. To start the reaction, (*6RS*)-5-CH_3_-H_4_folate was added. The total volume of the reaction was 800 µl. The reaction was quenched by adding 200 µl of a 13 M formic acid and 5 M hydrogen chloride mixture, followed by heating at 90°C for 10 min. After cooling down, absorbance at 350 nm was measured. To calculate the concentration of CH^+^ = H_4_folate, the molar absorption coefficient of 26.0×10^3^ was used 17]. Because we found high concentration of CH_3_-H_4_folate inhibited the enzyme activity (see Results), Equation 1 for substrate inhibition was used for fitting 18].
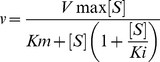
(1)


Values for *Km*, *Ki*, and *Vmax* were computed by KaleidaGraph (Synergy Software, Reading, PA).

### Molecular Mass Determination

Purified proteins were subjected to polyacrylamide gel electrophoresis under denaturing conditions using sodium dodecylsulfate (SDS-PAGE), according to the method of Laemmli 19]. The native molecular mass of Thermus MTHFR was determined by HPLC using a size exclusion TSK-gel G3000SWxl (Tosoh) column, and the protein was detected using a multi-angle light scattering detector (Dawn ESO (Wyatt Technology, Santa Barbara, CA)), and a refractive index detector (Shodex RI-71 (Showadenko, Tokyo, Japan)). Other parameters were as follows: mobile phase; 50 mM Tris-HCl buffer, pH 6.8, containing 0.2 M NaCl, flow rate; 0.5 ml/min, sampling; 0.1 mg protein/run.

### Anaerobic Titration

Anaerobic titration was done using an anaerobic cuvette and a Hamilton gastight syringe with a repeating dispenser 20]. Degassing of the protein solution and equilibration with Ar gas in the cuvette was performed using an anaerobic train. Spectra were recorded by the Cary spectrophotometer at room temperature.

### Protein Crystallization and data collection

The hanging drop vapor diffusion method was employed for protein crystallization. Drops were prepared by mixing equal volumes of a protein solution and a reservoir solution, 2 µl each for the as-purified MTHFR and 1.5 µl each for the FAD-replete protein. The concentration of purified His-tagged Thermus MTHFR was ≈20 mg/ml. Crystals suitable for data collection were obtained using a reservoir consisting of 0.1 M sodium acetate buffer (NaOAc, pH 4.3∼4.5), 1M lithium chloride, 10% polyethylene glycol (PEG) 6000, 10–20% glycerol, and 2–5% dioxane at 20°C for the as-purified MTHFR. For the crystallization of the FAD-replete MTHFR, the composition of the reservoir solution was 0.1 M Tris-HCl (pH 8.0), 0.2 M ammonium sulfate, 20–25% PEG 4000, and 5% glycerol. Crystals of the as-purified MTHFR put on a fiber loop were frozen in liquid nitrogen and thin crystals of the FAD-replete enzyme were retrieved by a micromesh. Then they were mounted under gaseous nitrogen. X-ray data sets were collected at the Photon Factory (Tsukuba, Japan) at the NE3 beam line equipped with a Quantum 270 CCD detector and the NW12A beam line equipped with a Quantum 210r CCD detector for the as-purified and the FAD-replete MTHFR crystals, respectively. The HKL2000 program suite was used for data processing 21].

### Phase Determination, Model Building, and Refinement

Thermus MTHFR crystals phases were determined by the molecular replacement method using the PDB file 1V93, the Thermus MTHFR monomer structure, as a model. The programs of MolRep 22] and Refmac5 23] in the CCP4i program suite were used for the molecular replacement and the model refinement, respectively. Manual corrections of coordinates were performed using the program COOT 24]. To generate figures the program PyMol 25] was used.

## Results

### Expression, Purification and Molecular Mass Determination

**Figure 2:**
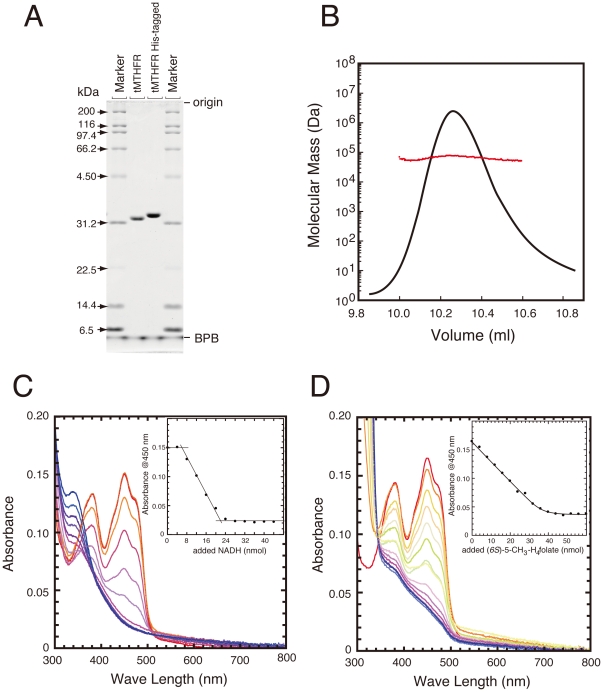
SDS-PAGE, Molecular Mass and anaerobic titration of Thermus MTHFR. A. Purity of Thermus MTHFR (tMTHFR) with or without a His-tag was analyzed by 12% polyacrylamide gel electrophoresis under denaturing conditions. The gel was stained by Quick-CBB (Wako Pure Chemical, Osaka, Japan). The molecular markers were obtained from Bio-Rad. BPB, bromophenol blue. B. Measurement of the native molecular mass of the as-purified MTHFR by size exclusion column chromatography using the multi-angle light scattering detector. The red dots show the molecular weight at each point in the chromatogram, while the solid line shows the elution profile detected by the refractive index detector. C. Anaerobic titration of the as-purified MTHFR with NADH. Fifteen nmol of enzyme (based on FAD content) was prepared in the anaerobic cuvette, then titrated with NADH. The initial spectrum is shown in the red line, and the final one is blue. (inset) Changes in absorbance at 450 nm. D. Anaerobic titration of the FAD-replete MTHFR with (*6RS*)- 5-CH_3_-H_4_folate. Fifteen nmol of enzyme was used. The initial spectrum is shown in the red line. The final spectrum, which is shown in blue, is resemble to the spectrum of the fully reduced FAD, indicating that (*6S*) - 5-CH_3_-H_4_folate could reduce all FAD to the fully reduced form of FAD. (inset) Changes in absorbance at 450 nm.

Purity of non-tagged wildtype and His-tagged wildtype Thermus MTHFR was examined by SDS-PAGE (Figure 2A). As judged on an SDS-PAGE gel, both preparations appeared homogenous and appropriate to examine biochemical properties and protein crystallization. Approximately 25 mg of purified protein could be prepared per L of culture. Preparations of highly purified enzyme were stable for at least 1 month at 4°C. For long term storage, concentrated samples were stored at −80°C. Since properties of wildtype and His-tagged wildtype MTHFR were essentially the same, the His-tagged wildtype enzyme was used for the following experiments unless otherwise noted. As shown in Figure 2A, the apparent molecular weight of Thermus MTHFR was estimated as 33 kDa, as predicted from DNA sequencing. The native molecular weight using the multi angle light scattering detector was calculated to be 66 kDa (Figure 2B), indicating that Thermus MTHFR is a dimer in solution.

### FAD Repletion

The absorption spectrum of the ‘as-purified’ MTHFR was typical of a flavoprotein, indicating that the purified protein bound FAD without any FAD treatment during expression and purification. We found that further addition of FAD to the purified enzyme solution could stimulate NADH:menadione oxidoreductase activity. These results indicated that the purified Thermus MTHFR was a mixture of holo- and apo-subunits, and the inactive apo-subunits were still able to bind FAD to form catalytically active subunits. The apparent *Km* for FAD of the apo-subunit was estimated to be ≈ 5 µM. Purified enzyme preparations were routinely activated 1.3-1.8 fold by the exogenous FAD. Therefore, FAD repletion in most preparations of the as-purified enzyme would be 55∼77% (≈60% on average), if the FAD treatment at the concentration of 5 mM was enough to bind all apo-subunits and the FAD repletion could be simply estimated from the stimulation of the enzyme activities. Enzyme tightly bound to the cofactor after the incubation with FAD, suggesting that the apo-subunit found in the as-purified MTHFR was not formed during purification steps. Heat-treatment was not necessary for the holo-enzyme formation. Since non-tagged enzyme could also be activated by additional FAD, the isolated apo-enzyme was not due to the His-tag on the C-terminus. Whereas exogenous FAD treatment was effective to form holo-enzyme without heat treatment after purification, the presence of riboflavin (≈12 µM), the precursor of FAD, in the expression medium did not affect FAD repletion of the enzyme. In addition, temperature dependency of FAD repletion during the protein expression was not observed.

### Spectral Properties and Anaerobic Titration

Anaerobic titrations of the as-purified MTHFR with NADH and the FAD-replete enzyme with CH_3_-H_4_folate are shown in Figure 2C and D, respectively. NADH could reduce bound FAD with approximately 1∶1 stoichiometry. For the titration of the oxidized FAD-replete MTHFR by CH_3_-H_4_folate, (*6RS*)-5-CH_3_-H_4_folate was used although only the *6S* isomer is active. The FAD cofactor could be fully reduced by CH_3_-H_4_folate (Figure 2D), indicating that the added FAD after purification was introduced into the active site in a proper orientation. For full reduction of the enzyme bound FAD, more than 2.6 equivalents of (*6S*)-5-CH_3_-H_4_folate were needed. Excess amounts of CH_3_-H_4_folate were required because of the equilibrium of CH_2_-H_4_folate/CH_3_-H_4_folate with reduced/oxidized FAD. Although results of other combinations, i.e. the FAD-replete MTHFR titrated with NADH and the as-purified enzyme with CH_3_-H_4_folate, are not shown, the observed stoichiometries of NADH/FAD and CH_3_H_4_folate/FAD are essentially identical to those shown.

### Temperature Dependency of Reaction and Specificity and Affinity for Substrates

**Figure 3:**
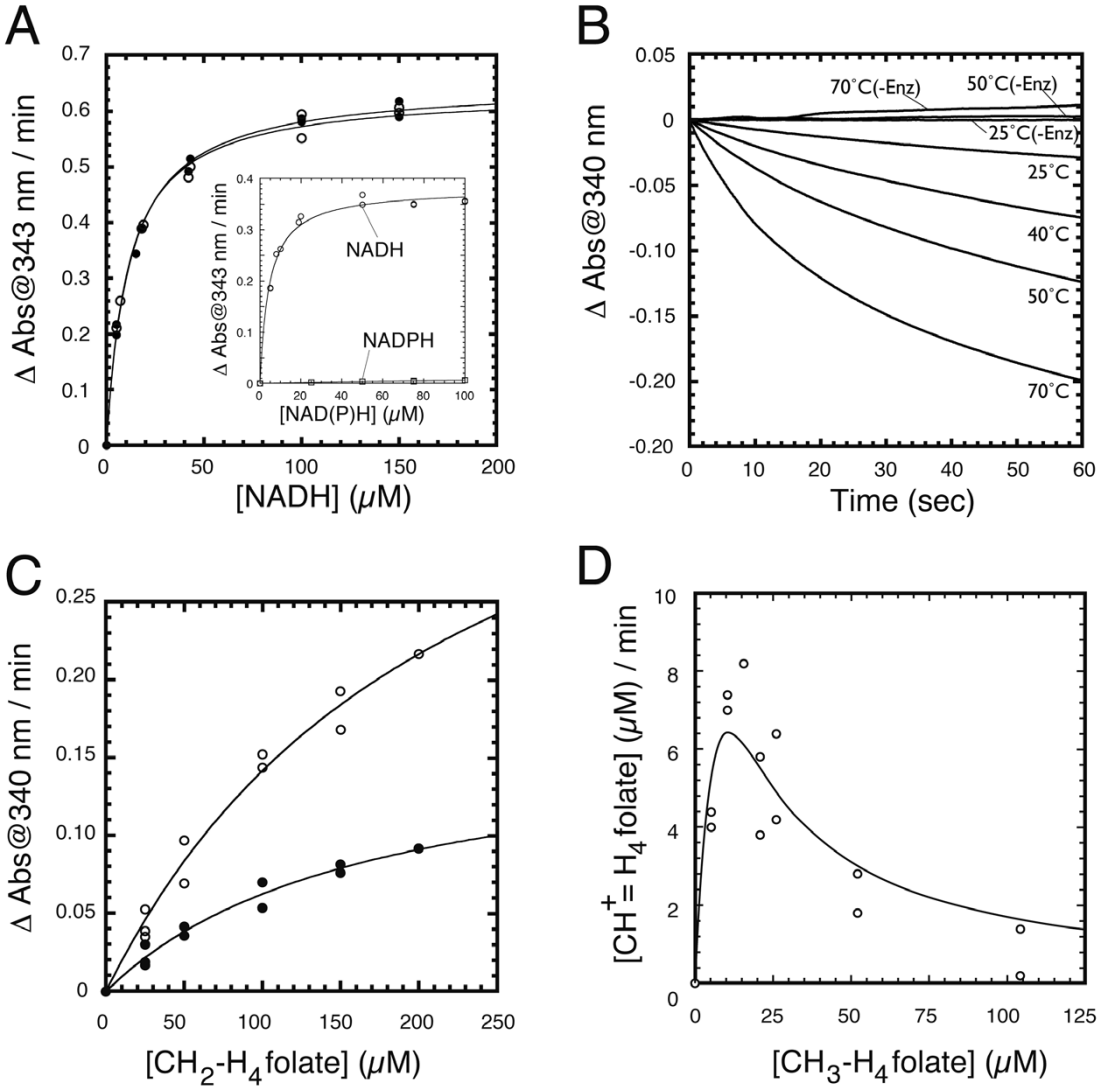
Catalytic properties of Thermus MTHFR. A. NADH:menadione oxidoreductase activity of the as-purified (open circle) and the FAD-replete MTHFR (filled circle). Enzyme activities were determined with varying amounts of NADH. In this comparison, the same amounts of FAD bound subunits ([Et]) were used. (inset) Comparison of the enzyme activities using NADH and NADPH. NADH is a better substrate than NADPH for Thermus MTHFR. B. Temperature dependence of NADH: CH_2_-H_4_folate oxidoreductase activity using the as-purified MTHFR. Consumption of NADH was monitored for 1 min. Changes in absorbance measured without enzyme are expressed as “-Enz”. C. NADH: CH_2_-H_4_folate oxidoreductase activity of the as-purified (open circle) and the FAD-replete MTHFR (filled circle). The same amount of [Et] was used for assays of the as-purified and replete enzymes at 50°C. D. Substrate inhibition in CH_3_-H_4_folate:menadione oxidoreductase activity. The as-purified MTHFR was used for the assay and the activities were determined at various concentrations of (*6S*)-5-CH_3_-H_4_folate (up to 104 µM). The enzyme product, CH_2_-H_4_folate, was determined after conversion to CH^+^ = H_4_folate.

Kinetic parameters of the as-purified MTHFR based on steady-state kinetic analysis are shown in Table 1. The NADH:menadione oxidoreductase activity of Thermus MTHFR was reasonably high at room temperature. As with *E. coli* MTHFR 13], Thermus MTHFR preferentially used NADH rather than NADPH (Figure 3A, inset). In contrast to the reductive half reaction, high temperature was needed to measure NADH:CH_2_-H_4_folate oxidoreductase activity (Figure 3B). The enzyme activity was high at 70°C, which is an optimum growth temperature for this bacterium 26]. It is technically quite difficult to determine the enzyme activities by the spectrophotometric assay with accuracy at such high (≈70°C) temperature. We decided to measure the enzyme activities at 50°C when folate-derivatives were used as substrates. It was reported that *E. coli* MTHFR showed substrate inhibition for both NADH and CH_2_-H_4_folate. Substrate inhibition, however, was not apparently observed when activities of Thermus MTHFR were measured at up to 150 µM NADH and 200 µM CH_2_-H_4_folate (Figure 3A and C, respectively).

**Table 1:** Steady-state kinetic parameter of Thermus MTHFR^1.2^.

	Vmax/[Et]	Affinity for substrates	
Assay	(min^−1^)	substrates	affinity
NADH:CH_2_-H_4_folate oxidoreductase activity (at 50°C)	290±43	CH_2_-H_4_folate^3^	180±23 µM *(Km)*
NADH: menadione oxidoreductase activity (at 25°C)	3700±280	NADH	9.7±2.1 µM *(Km)*
CH_3_-H_4_folate: menadione oxidoreductase activity (at 50°C)	600±170	CH_3_-H_4_folate^4^	17±1.2 µM *(Km)*
		CH_3_-H_4_folate^4^	15±6.0 µM *(Ki)*

1 Values are expressed as average ± SE from three independent assays

2 The as-purified MTHFR was used for all assays.

3 (*6R)*-5,10-CH_2_-H_4_folate was prepared by the non-enzymatic reaction of (*6S*)-H_4_folate and formaldehyde.

4. Affinities for (*6S*)-5-CH_3_-H_4_folate.

In contrast to the two other assay methods, substrate inhibition was observed in the CH_3_-H_4_folate: menadione oxidoreductase activity. Figure 3D shows a plot of CH_3_-H_4_folate: menadione oxidoreductase activity of the as-purified enzyme as a function of substrate concentration. Equation 1 (see Methods) for substrate inhibition was used for fitting the plot. As shown in Table 2, *Km* and *Ki* values were estimated to 17 µM and 15 µM, respectively, indicating that CH_3_-H_4_folate could act as a very strong inhibitor of the enzyme. Although data are not shown, the FAD-replete enzyme also showed substrate inhibition. The distinct property of the substrate inhibition of the enzyme by CH_3_-H_4_folate suggest some unique role of MTHFR in this thermophilic organism.

**Table 2:** Data collection and refinement statistics.

	As-purified MTHFR	FAD-replete MTHFR
Wavelength Å	1	1
Reflections	422075	215954
Unique reflections	55102	57531
Completeness (%)	99.54	93.85
R_sym_ a	0.078	0.093
I/σ(I)	53.3	14.3
Space group	*P*2_1_2_1_2_1_	*P*2_1_
Cell (Å)	*a* = 43.92, *b* = 89.67, *c* = 160.72	*a* = 116.59, *b* = 90.93, *c* = 125.15
	*β* = 90°	*β* = 90°
Resolution (Å)	20 - 1.85	20 – 2.80
R_cryst_ b/R_free_ c	0.204/0.254	0.209/0.298
Average B-factor	36	19.5
RMSD bonds (Å)	0.028	0.016
RMSD angles (deg)	2.338	1.741
Ramachandran plot		
most favored (%)	96.6	96.5
additional allowed (%)	2.3	2.8

a R_sym_  =  Σ*_hkl_*Σi|*I_i_(hkl)-I(hkl)*|/Σ_hkl_Σ*Ii(hkl)*, where *Ii(hkl)* is the *i*th intensity measurement of reflection hkl, including symmetry related reflections, and *I(hkl)* is its average.

b R_cryst_  =  Σ_hkl_ (|Fo|-|Fc|)/Σ_hkl_|Fo|.

c R_free_ was calculated on 5% of the data omitted randomly

While FAD-repletion of Thermus MTHFR showed no effect on NADH:menadione oxidoreductase activity calculated on a per-flavin basis (Figure 3A), the FAD-replete MTHFR showed low NADH: CH_2_-H_4_folate oxidoreductase activities when the same [Et] (total enzyme bound flavin) was used (Figure 3C). The enzyme activity of the FAD-replete enzyme was approximately 50% of that of the as-purified MTHFR. Similarly, the CH_3_-H_4_folate:menadione oxidoreductase activity of the FAD-replete MTHFR was also reduced to ≈60% of that of the as-purified enzyme (data not shown). These observations could be simply explained if both subunits had ≈50% activities or if one of two FAD-binding subunits had little or no activity for the folate reaction. If actions of the one subunit were diminished by the other subunit, this cooperativity could be interpreted as half-of-the-site reactivity.

### Thermal and pH Stability

**Figure 4:**
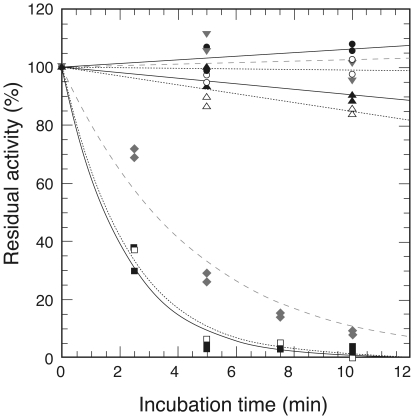
Thermostability of Thermus MTHFR. Thermostability of the as-purified (open symbols with dotted lines) and the FAD-replete MTHFR (filled symbols with solid lines). Diluted enzymes ([Et]  =  2 µM) were incubated at 70°C (circle), 80°C (triangle), and 90°C (square) for the indicated times, then put on ice. The symbols of grey-colored triangles and diamonds with dashed lines represent the FAD-replete MTHFR with 10 µM FAD at 80 and 90°C, respectively. Enzyme activities were measured by NADH:menadione oxidoreductase assay.

Thermostability of the as-purified MTHFR and the FAD-replete enzyme was examined (Figure 4). MTHFR from the thermophile was totally stable at 70°C. Although very minor heat inactivation was observed at 80°C, addition of free FAD (10 µM) could completely rescue the enzyme from inactivation. At 90°C, half of the enzyme was inactivated within two minutes, and free FAD could partially alleviate inactivation. These properties of the as-purified MTHFR and the FAD-replete enzyme were indistinguishable, indicating that the treatment with FAD after purification did not affect the thermostability.

Since the pH of the 0.1 M NaOAc buffer used in the crystallization was as low as 4.3, the effect of pH on stabilities of apo-subunits was examined. After the as-purified enzyme (10 µM) was incubated with 0.1 M NaOAc buffer pH 4.3 containing 0.1 M NaCl for 2 days at 4°C, FAD was added to the solution. Then, enzyme activities were determined by NADH:menadione assay. Approximately 80% activity was observed after the 2-day incubation, suggesting that the apo-subunit was reasonably stable even in dilute solution at the low pH.

### Crystal Structures of Thermus MTHFR

**Figure 5:**
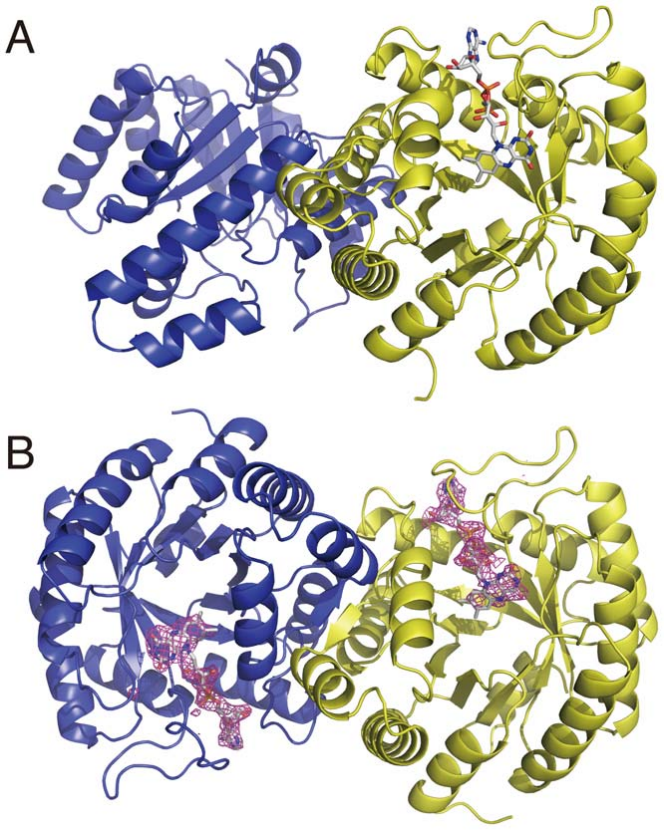
The dimers of the as-purified and the FAD-replete Thermus MTHFR. A. The structure of the crystal of the as-purified MTHFR is shown in ribbon mode. The yellow colored subunit is the holo-subunit, which contains FAD drawn in stick mode. The blue colored subunit represent the apo-subunit that lacks the FAD cofactor in the active site. B. Ribbon drawing of the fold of the FAD-replete MTHFR. The dimer contains one FAD molecule in each subunit. FAD found in the subunit is drawn in stick mode along with the electron density from an omit map (|Fo|-|Fc| map, 3.0σ) computed after refinement.

**Figure 6:**
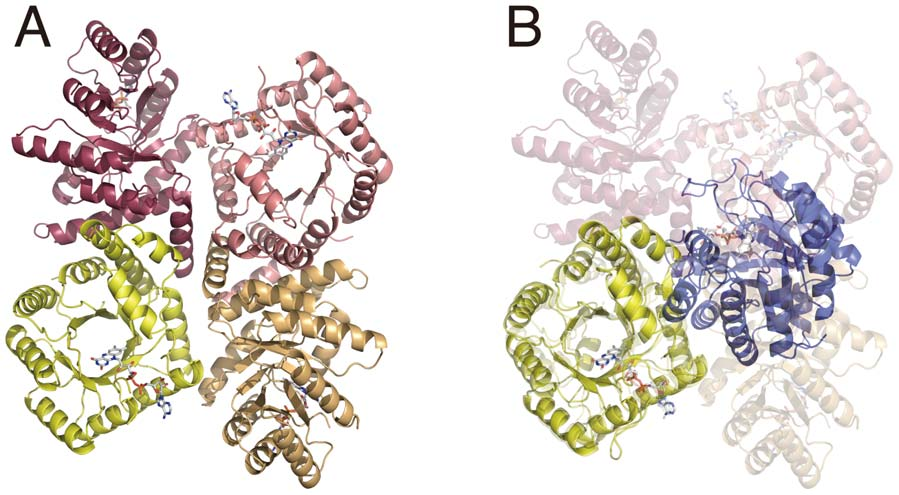
Comparison of quaternary structures of *E. coli* and Thermus MTHFRs. A. A tetramer of *E. coli* MTHFR, viewed down the local two-fold axis, is drawn using the PDB file (1ZPT) as determined by Pejchal *et. al.* (*Biochemistry* (2006) 45, 4808-4818). B. A dimer of FAD-replete Thermus MTHFR is shown overlaid on *E. coli* MTHFR (transparent). Superimposed subunits are colored in yellow for both MTHFRs. It is clear that none of subunits of *E. coli* MTHFR can overlap the blue-colored subunit of the Thermus enzyme.

Overviews of the dimers of the as-purified and the FAD-replete MTHFR are shown in Figure 5A and B, respectively. Crystal data and statistics are shown in Table 2. Crystals of the as-purified MTHFR contained a dimer comprising apo- and holo-subunits (see below). This was expected from activity measurements that had predicted the apo-subunit in the purified protein. The FAD-replete MTHFR in crystals was a homodimer of two holo-subunits (Figure 5B). FAD treatment after purification not only contributed to the holo-subunit formation, but also dramatically altered the subunit contacts in the dimer. The large conformational change resulted from an ≈50° rotation of the apo-subunit relative to the holo-subunit upon FAD-binding. Comparison of the intermolecular interface of the as-purified and the FAD-replete MTHFR is shown in Figure S2. While the crystallization conditions were different, the conformational change of Thermus MTHFR is likely occurred by the ligand binding rather than by effects of hydrophobic solvent and the low pH, which we used when crystallization of the heterodimer (See detail in Figure S2). In the holo-dimer structure, a β-sheet was newly formed at the intermolecular interface, which was designated as β7'. The secondary structure is illustrated in Figure S3. The *β_8_α_8_* topology of the holo- subunit barrel of Thermus MTHFR is quite similar to that of holo-subunits *E. coli* MTHFR, which is a homotetrameric protein in solution. Figure 6 shows a comparison of quaternary structures of *E. coli* and Thermus MTHFR. Despite the resemblance of their subunits, the intermolecular interface of *E. coli* MTHFR had no similarity to that of Thermus enzyme, indicating that the subunit interfaces of MTHFRs may be diverse. While the FAD-replete Thermus MTHFR was used to illustrate Figure 6 for comparison, the subunit interface of the as-purified enzyme was different from that of *E coli* enzyme as well (data not shown). Because the Thermus MTHFR shares dimeric symmetry with the mammalian enzyme, it may be a better model for the conformational changes associated with FAD loss in the mammalian enzyme.

### Structure of As-purified Thermus MTHFR

**Figure 7:**
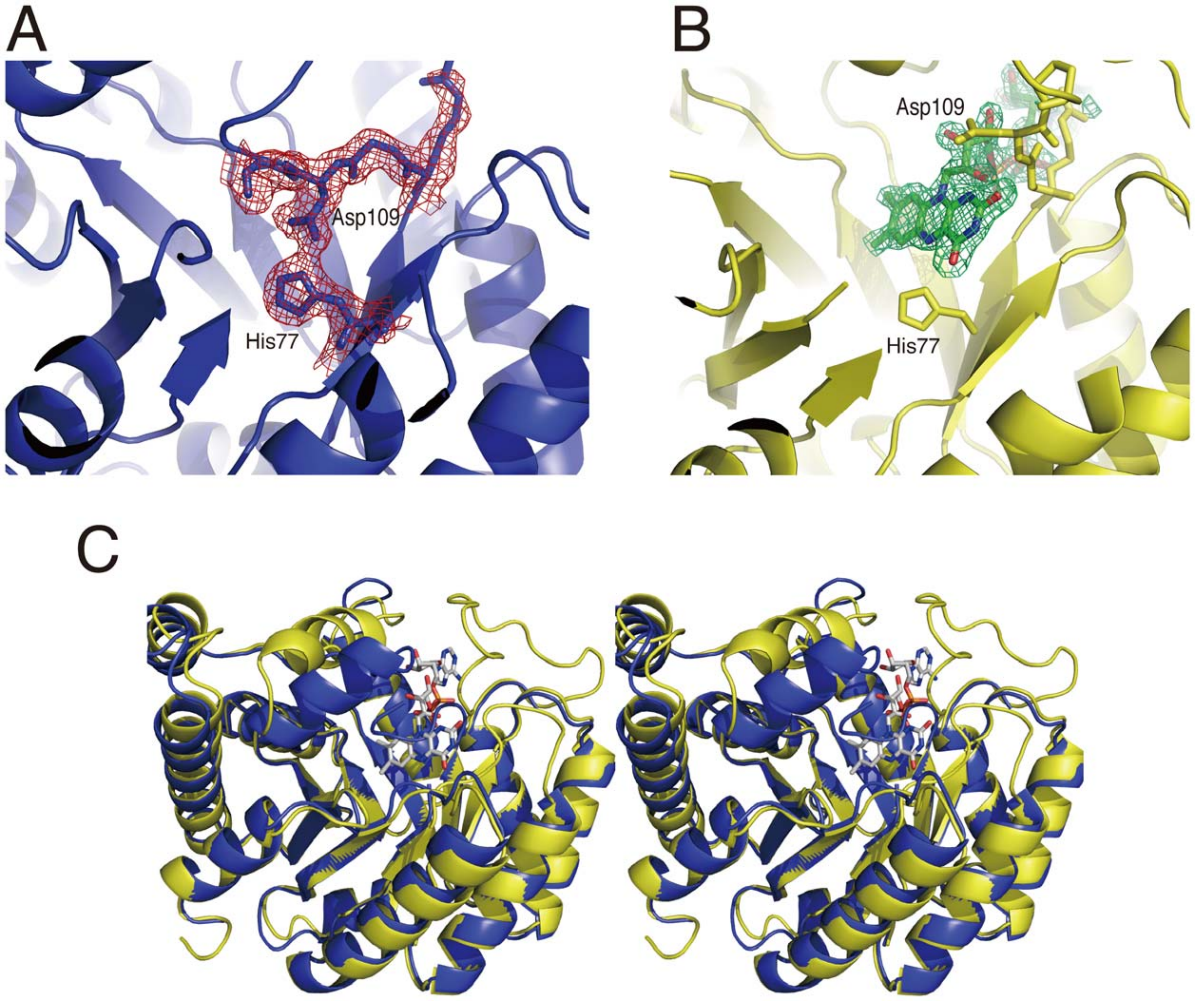
Comparison with the apo- and holo-subunits of the as-purified enzyme. A. Ribbon drawing of the center of the *β_8_α_8_* barrel (the apo-subunit) is shown. Five amino acid residues (His77, Arg107, Gly108, Asp109, and Pro110) are shown in stick mode with a 2|Fo|-|Fc| map (1.0 σ). His77 interacts with Asp109. B. The active center of the holo-subunit is drawn in ribbon mode but five amino acid residues (His77, Arg107-Pro110) are shown in stick mode. FAD is shown in stick mode with an omit map (4.0 σ). The position of Asp109 is markedly changed by binding of FAD. C. A stereo view showing superimposition of the apo- and holo-subunits of the as-purified MTHFR dimer. Protein folding is drawn in ribbon mode. The FAD cofactor is shown in stick mode. The color scheme is the same as in the Figure 5A.

The FAD cofactor has a tricyclic heteronuclear ring, the isoalloxazine ring. Although the functional bulky group should be easily found during model building and refinement, preliminary analysis of the initial |Fo|-|Fc| map indicated that only one tricyclic ring could be found per dimer. This was in good agreement with the result obtained from ≈60% FAD repletion of the preparation. This crystal form was, therefore, considered to be a heterodimer of holo- and apo-subunits. Figure 7B and 7A show the active sites of the holo- subunit and the corresponding position of the apo-subunit, respectively. This is the first determination of the apo-subunit of MTHFR, whereas the structure of the holo-subunit was almost identical to previously solved *E. coli* 5] and Thermus MTHFR structures (PDB 1V93). Because of the fair stability of the apo-subunit at pH 4.3, the structure of the apo-subunit could be considered as one possible structure of MTHFR. The intersubunit interface, which was different from those in the *E. coli* MTHFR structure 5] 27], was only ≈900 A^2^ and was 6.4–7% of the total molecular surface of the subunit in the crystal. Nevertheless, despite the small area of the intersubunit surface, as-purified Thermus MTHFR was shown to be a dimer in solution by size exclusion chromatography.

Superimposition of the holo- and the apo-subunits is illustrated in Figure 7C. In the holo-subunit, the FAD cofactor bound to the active center. Near the *si*-face of the isoalloxazine ring of FAD, the catalytically essential Glu18 was found. In the active site of *E. coli* MTHFR, Glu28 and Asp120 near the isoalloxazine ring of FAD were identified as catalytically important residues 6]; they correspond to Glu18 and Asp109 in Thermus MTHFR, respectively. Asp109, whose proposed function is stabilizing the 5-iminium cation form of CH_2_-H_4_folate when the substrate was bound, is positioned near the C2-position of the isoalloxazine ring (Figure 7B) 7]. In the apo-subunit, positions of the loop containing Asp109 and helix *α*7a are dramatically different, although the topology of the *β_8_α_8_* barrel of the apo-subunit is almost identical to that of the holo-subunit. (Figure 7C) Asp109 in the apo-subunit interacts with His77 (Figure 7A), which is close to the N5-position of the isoalloxazine ring of FAD in the holo-subunit (Figure 6B). As a result, the center of the *β_8_α_8_* barrel of the apo-subunit is completely buried with the loop and helix (*α*7a), whereas the corresponding site of the holo-subunit is widely opened

### Structure of FAD-replete Thermus MTHFR

**Figure 8:**
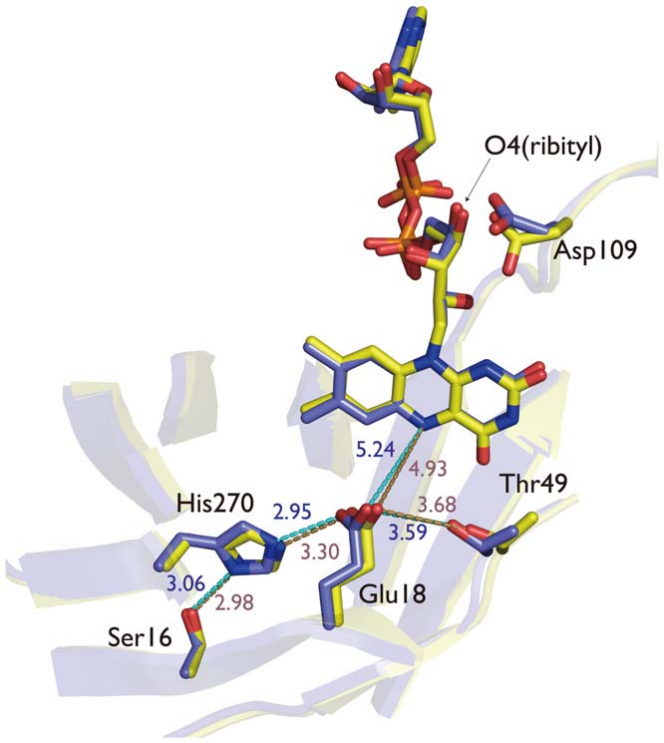
Superimposition of the holo-subunits of the FAD-replete MTHFR. Structures of the active sites of both holo-subunits of the dimer are compared. Positions of Glu18 and Asp109, which are homologous to catalytically important amino acid residues in the *E. coli* enzyme, are especially focused. Distances (Å) between atoms are also shown in brown and blue for the yellow and blue subunits in Figure 5B, respectively. Distances from O4 of ribityl chain of FAD to Oε of Asp109 were 2.58 and 2.71 Å for the yellow and blue subunits, respectively.

As shown in the Figure 5B, the FAD-replete MTHFR has one FAD cofactor per subunit, forming a dimer of holo-subunits. A non-crystallographic two-fold axis is found between the *β*7' strands. Both FAD cofactors in the dimer showed NADH:menadione oxidoreductase activity as expected. Furthermore, CH_3_-H_4_folate could reduce the enzyme bound FAD completely (Figure 2D). Therefore, at least, the flavin introduced after purification binds in the proper orientation. In fact, it was impossible to distinguish which subunit was originally the apo-subunit when the enzyme was purified. However, the enzyme showed only 50% activity with folate substrates in steady state kinetics. Superimposition of the active site of the holo-subunits is illustrated in Figure 8, particularly focused on Glu18 and Asp109, since their importance is proposed by the *E. coli* MTHFR study 6]. In the FAD-replete MTHFR that was determined from crystals grown at pH 8.0, distances of Glu18(Oε)- His270(Nε), His270(Nδ)- Ser16(Oγ), and O4(ribityl chain of FAD)- Asp109 (Oε) were estimated as 2.98–3.06 Å, 2.95–3.30 Å and 2.58–2.71 Å, respectively (Figure 8). In the holo-subunit of the as-purified MTHFR, corresponding distances were 2.83 Å, 2.68 Å and 4.37 Å, respectively, where the structure was determined from crystals at acidic pH (≈4.4). The diminished folate reaction in the FAD-replete enzyme is described greater detail in Discussion.

## Discussion

In the absence of a crystal structure for human MTHFR, the *E. coli* enzyme has previously served as a model for the structural and biochemical characterization of this family of enzymes. In particular, the structure of the *E. coli* enzyme has suggested a mechanism by which the 677C→T polymorphism in human MTHFR that substitutes Ala222 by Val might lead to enhanced propensity for the dimeric enzyme to release its flavin cofactors and dissociate into monomers 5] 27]. Our studies of the MTHFR from *T. thermophilus* have provided additional insights into the linkage between dissociation of the dimer into monomers and flavin release. We have shown that the enzyme undergoes a dramatic conformational change when flavin is absent, a change that alters the intersubunit packing of the dimer and that is expected to alter the catalytic activity of the enzyme. Changes in subunit packing may also occur when human MTHFR loses its flavin, and/or when the enzyme is allosterically inhibited by AdoMet. **The significance of these findings is discussed in detail below**

### 

#### Thermus MTHFR as a model for human MTHFR

From sets of our experimental data, the as-purified MTHFR could be defined as a ‘heterodimer’ comprising apo- and holo- subunits, and the FAD-replete enzyme as a ‘homodimer’ formed by the two holo-subunits. Although *E. coli* MTHFR is a tetramer of four holo-subunits 13], MTHFR from native cells of *P. productus* was reported to be an octamer of 32 kDa subunits containing 4 FAD per octamer 14]. Similarly, the heterodimer of Thermus MTHFR may be the physiological form, since it is maximally active in catalyzing the physiological reaction.

**Figure 9:**
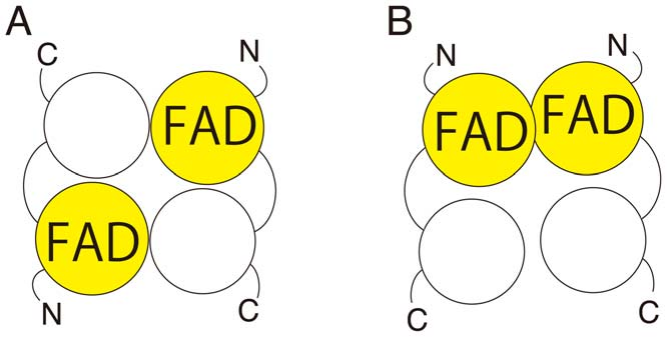
Possible models for mammalian MTHFR dimer. For a schematic model of mammalian MTHFR, catalytic and regulatory domains are shown in yellow with FAD and white, respectively. Using *E. coli* MTHFR as a model, mammalian MTHFR can be illustrated as in A, because the tetramer of *E. coli* MTHFR contains the local two-fold axis in the center of the tetramer (Figure 6A). Thermus MTHFR structure predicts the different model shown in B, in which the catalytic domains interact ‘side-by-side’.

Mammalian MTHFR is a dimeric protein with each monomer consisting of two discrete domains (catalytic and regulatory). Despite having only catalytic domains, *E. coli* MTHFR has been offered as a model for human MTHFR studies 5] 27]. This is because the tetrameric *E. coli* MTHFR resembles the planar rosette of porcine MTHFR observed by scanning transmission electron microscopy 28] (Figure 9A). The actual subunit interface of mammalian MTHFR, however, has not yet been determined. A possible alternative model of mammalian MTHFR based on the Thermus MTHFR structure is illustrated in Figure 9B. Based on the previous model using *E. coli* MTHFR (Figure 6A), the catalytic subunits are placed on the diagonally opposite side and contact each other with no or quite low surface area. The Thermus MTHFR structure would predict direct interaction between the catalytic domains in the dimer as shown in Figure 9B.

#### Reduced catalytic function on folate-dependent reaction on the homodimer

While the catalytic efficiency for the folate-dependent reaction of the homodimer is decreased to ∼50%, we observed that all flavin cofactors bound to the enzyme could be reduced by CH_3_-H_4_folate by the anaerobic titration. Figure 8 compares the active sites of the two subunits of the homodimer. Two amino acid residues, Asp119 and Glu28, are important for the catalytic function of *E. coli* MTHFR 6]. They are conserved in all MTHFRs and correspond to Asp109 and Glu18 in Thermus MTHFR, respectively. Thus, we assume that the chemical principles in the reaction of Thermus MTHFR are analogous to those of *E. coli* MTHFR. The substitution of the Glu28 of *E. coli* MTHFR by Gln completely abolishes the folate-dependent reaction and also dramatically reduces the NADH-dependent FAD reduction 29]. Currently, the function of the Glu residue is proposed to be stabilizing the 5-iminium cation of the folate intermediate and/or serving as the proton donor for CH_2_-H_4_folate to form the 5-iminium cation. Though the Glu residue could be responsible for the reduced function of the homodimer of Thermus MTHFR, the NADH-dependent reaction was not affected by homodimer formation.

Asp109 is placed above N1-C2 = O of the isoalloxazine ring of FAD. The Asp residue is crucial for binding of folate and it stabilizes the 5-iminium cation form of the substrate. Trimmer et al 7] have reported that the substitution of the Asp residue of *E. coli* MTHFR by Asn greatly reduces the catalysis of the folate-dependent reaction but not the NADH-dependent FAD reduction. In *E. coli* MTHFR, distances between O4 of the ribityl chain of FAD and Oε of Asp119 varied under different conditions: it was 3.63–4.60 Å when the enzyme was substrate free at pH 6.0 (PDB 1B5T), 2.59–2.66 Å when NADH was bound at pH 7.3 (PDB 1ZPT), 3.98–4.22 Å when CH_3_-H_4_folate was bound at pH 7.4 (PDB 1ZP4). In our Thermus MTHFR structure, the Asp residue was 2.58–2.71 Å apart from the O4 of FAD in the ligand-free homodimer at pH 8.0, thus it was located in similar position of that of the NADH binding form of *E. coli* MTHFR. Since the lowered activities of Thermus MTHFR were exclusively found in the folate-dependent reactions, the positioning of Asp109 could be responsible. Our biochemical and structural analysis in this study, however, could not clarify whether the ‘half-of-the-site reactivity’ is an appropriate description of the properties of the homodimer. Additional studies, such as rapid kinetic analysis and structural determination with folate derivatives or analogues, would contribute to further understanding of the enzyme properties.

#### Two different intermolecular interfaces of Thermus MTHFR as a model for the allosteric regulation of human MTHFR, and possible relevance to the properties of the Ala222Val common mutant

Although it is not clear why the heterodimer is formed in *E. coli* cells, we have observed two different intersubunit interfaces of Thermus MTHFR. The conformational change could result in modulation of the catalytic function of the enzyme (see above). Similar changes in subunit packing might underlie the allosteric regulation of human MTHFR by AdoMet. The low surface area found in the intersubunit interface of the heterodimer of Thermus MTHFR would resemble the conformation of human MTHFR without AdoMet, and the homodimer could simulate the AdoMet binding form of human MTHFR.

The molecular mechanism of the allosteric regulation of human MTHFR remains largely uncertain. This is because crystallization of recombinant human MTHFR has not yet succeeded despite efforts in several laboratories. It is quite challenging to discuss the mechanism without any structural models. The subunit of human enzyme consists of catalytic and regulatory domains. The catalytic domain should be similar to the *β_8_α_8_* barrel of bacterial MTHFRs, because of the high homology. To control the activity by the allosteric inhibitor, the protein has to transfer the signal of binding of AdoMet, which should bind outside of the barrel, to the active center. Mammalian MTHFR induces a large conformational change upon AdoMet binding and enzyme activity is lowered to 10-20% 28]. We observed modulation of Thermus MTHFR activity by such a conformational change. This suggests that the shifting orientation of subunits is a possible mechanism for the allosteric inhibition of mammalian MTHFR.

This model for the conformational change of mammalian MTHFR by AdoMet might also explain the protective effect of AdoMet on the FAD loss of Ala222Val mutant on dilution. In the absence of AdoMet, Ala222Val mutant releases FAD faster than wildtype enzyme. Activity of the Ala222Val mutant is also inhibited by AdoMet, but the rate of flavin loss is decreased in the presence of AdoMet 11], suggesting that the conformational change could prevent dimer dissociation. Applying the Thermus MTHFR as the model, the properties might be explained, as follows: the low intersubunit contact area in the ligand free form would contribute to the lesser stabilization, which should be enhanced by the mutation, and the increased area of the interface in the AdoMet-binding form would support stronger interaction between subunits to stabilize the enzyme. After dissociation of the dimer followed by FAD loss, the apo-subunit of human MTHFR would be irreversibly denatured. Free FAD in solution, therefore, can prevent the enzyme from inactivation 11]. This hypothesis should be further examined in future studies.

In summary, properties and crystal structures of Thermus MTHFR are reported for the first time for a thermophilic MTHFR. The thermostable dimeric protein could be an alternative model other than *E. coli* MTHFR, which is a tetramer. The unique dimer of the Thermus MTHFR could form either heterodimer or holodimer. We found that binding of FAD to Thermus MTHFR could alter the reactivity to folate substrates accompanied by a large conformational change. The modulation of enzyme activities by altering a subunit interface might be related to the allosteric regulation of human MTHFR, which is also a dimer. In addition, a possible explanation for properties of the MTHFR mutant caused by the 677C→T common polymorphism was discussed using this bacterial enzyme as the model.

#### Coordinates

The coordinates have been deposited in the Protein Data Bank (Accession codes 3APT and 3APY for the heterodimer and homodimer, respectively).

## Supporting Information

Figure S1 **DNA and deduced amino acid sequences of pET(tMR^wt^-H).** The pET(tMR^wt^-H) vector was constructed to express Thermus MTHFR with the hexa-His tag on C-terminus. Gene specific primers used for PCR amplification are underlined. The original stop codon was mutated to Ala, which is shown in red.
(TIF)

Figure S2 **Comparison of the intermolecular interface of the as-purified and the FAD-replete MTHFR.** Color scheme is the same as in Figure 5. A) The structure of the as-purified (heterodimer) MTHFR (at pH∼4.4) is shown in the upper panel. The intermolecular interface (the boxed portion in the upper panel) is rotated anti-clockwise (90°) and illustrated in the lower panel. The intermolecular interface of the as-purified (heterodimer) MTHFR is small. Hydrophobic residues (Phe189, Tyr188, and Leu192) on the helix 6 form a hydrophobic area. Formation of a salt bridge between Arg179 and Glu267 is suggested by their distance. Asn184 and Glu263 could interact by hydrogen bond. B) The structure of the FAD-replete (homodimer) MTHFR (at pH 8.0) and its vertically rotated (90°) structure are illustrated in the upper and lower panels, respectively. Subunits in the FAD-replete (homodimer) MTHFR are interacting with larger area than those of the as-purified (heterodimer) protein. Two salt bridges, Arg197-Glu193 and Arg218-Glu237, can be found on the protein surface. We propose that the conformational change is likely due to the ligand (FAD) binding, rather than the hydrophobic reagent (dioxane) and low pH, by following reasons. Crystals of the as-purified MTHFR could be obtained without the hydrophobic reagent (dioxane). Determined models form crystals with or without dioxane are the same, indicating that the reagent should not be related to the conformational change. On one hand, Arg197 forming salt bridge with Glu193 in the FAD-replete (homodimer) MTHFR, on the other hand, a salt bridge between Arg197 and Glu267 could be formed in the as-purified MTHFR. Given the low pH (at pH∼4.4, we used to obtain crystals of the as-purified MTHFR) could disrupt the salt bridge of Arg197-Glu193, the same amino acid residue would not form salt bridge with other Glu residues. Both Glu193 and Glu 267 are on protein surface without any specific interaction to other residues. This suggests that the structure of the as-purified MTHFR is not stabilized by the low pH. In addition, we demonstrated stability of the heterodimer at pH 4.3 (see text). The acid-stable form is not the stabilized one at low pH. Thus, it would be quite rational that we obtained two different crystals from two forms of the protein in solution.
(TIF)

Figure S3 **Alignment of the deduced amino acid of MTHFRs.** Alignment of the deduced amino acid of MTHFRs from *T. thermophilus* HB8, *E. coli* K12, and human (the catalytic domain) is shown. The secondary structure of both bacterial MTHFRs is illustrated in this figure. Assignment of secondary structure of *E. coli* MTHFR was cited from Pejchal *et. al.* (*Biochemistry* (2005) 44, 11447-11457). Conserved amino acids between these three species are highlighted by yellow. For Thermus MTHFR, amino acid residues involved in the intermolecular interface of either hetero- or homo-dimer are colored in cyan. In *E. coli* MTHFR, the amino acid residues found at the interface are also shown in cyan. Amino acid residues forming the intermolecular interface were not conserved between *E. coli* and *Thermus* MTHFR.
(TIF)
