# Supplementary figures and images for: Properties and Crystal Structure of Methylenetetrahydrofolate Reductase from Thermus thermophilus HB8
Source: PLoS One. 2011 Aug 15;6(8):e23716. doi: 10.1371/journal.pone.0023716 (PMC3156243; doi:10.1371/journal.pone.0023716)

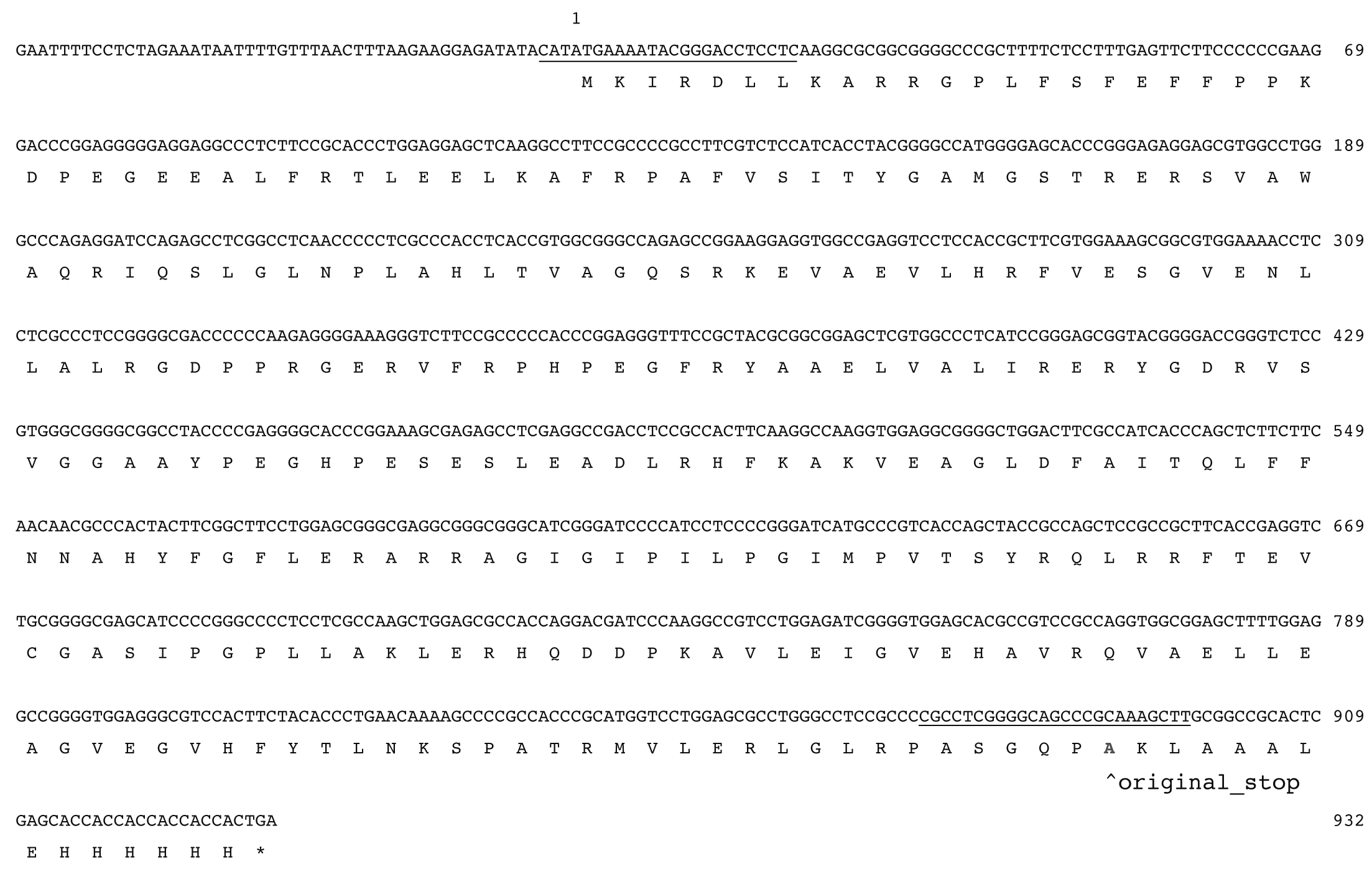

Supplement: Figure S1 — DNA and deduced amino acid sequences of pET(tMRwt-H). The pET(tMRwt-H) vector was constructed to express Thermus MTHFR with the hexa-His tag on C-terminus. Gene specific primers used for PCR amplification are underlined. The original stop codon was mutated to Ala, which is shown in red. (TIF) [file pone.0023716.s001.tif]

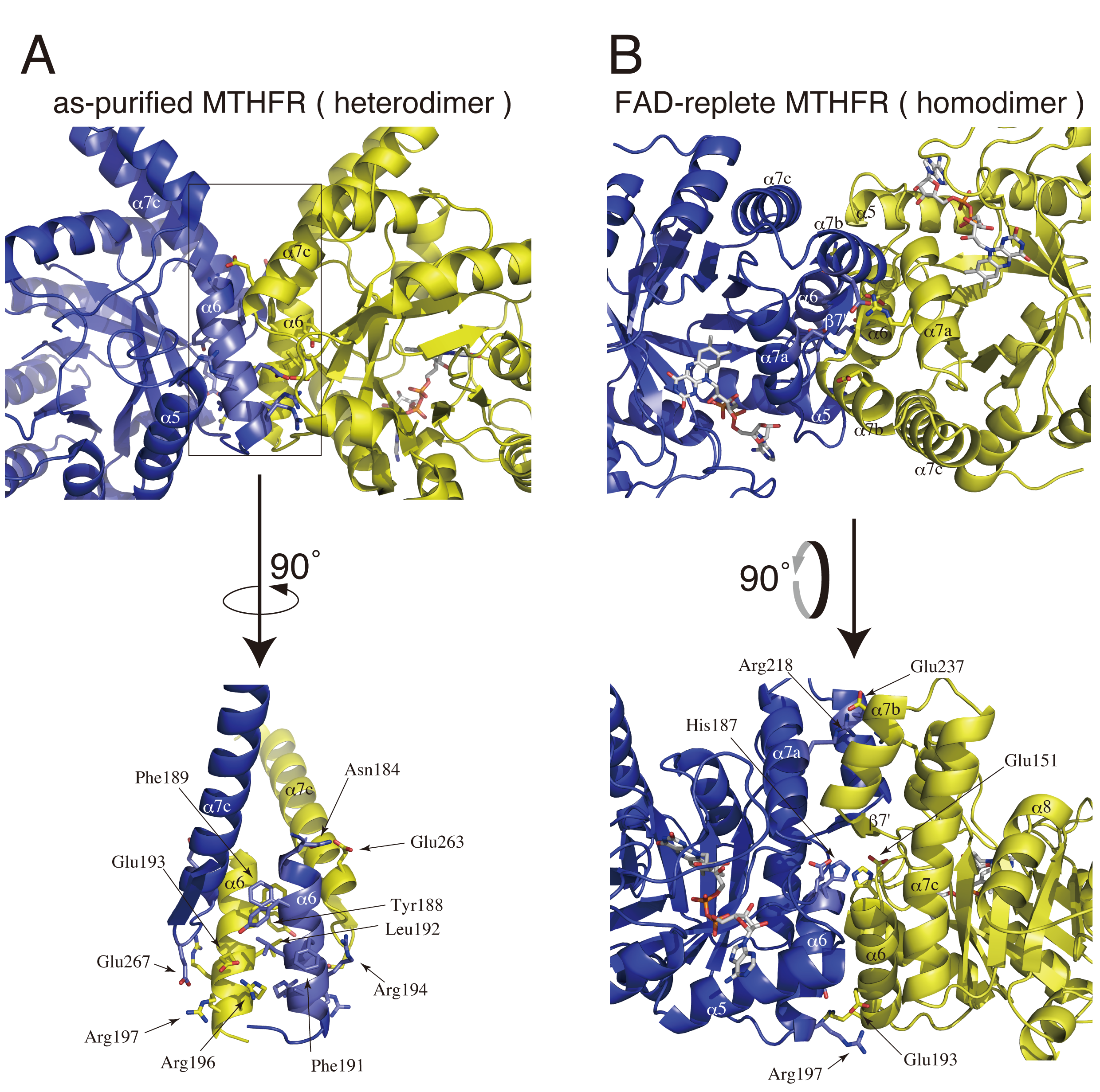

Supplement: Figure S2 — Comparison of the intermolecular interface of the as-purified and the FAD-replete MTHFR. Color scheme is the same as in Figure 5. A) The structure of the as-purified (heterodimer) MTHFR (at pH∼4.4) is shown in the upper panel. The intermolecular interface (the boxed portion in the upper panel) is rotated anti-clockwise (90°) and illustrated in the lower panel. The intermolecular interface of the as-purified (heterodimer) MTHFR is small. Hydrophobic residues (Phe189, Tyr188, and Leu192) on the helix 6 form a hydrophobic area. Formation of a salt bridge between Arg179 and Glu267 is suggested by their distance. Asn184 and Glu263 could interact by hydrogen bond. B) The structure of the FAD-replete (homodimer) MTHFR (at pH 8.0) and its vertically rotated (90°) structure are illustrated in the upper and lower panels, respectively. Subunits in the FAD-replete (homodimer) MTHFR are interacting with larger area than those of the as-purified (heterodimer) protein. Two salt bridges, Arg197-Glu193 and Arg218-Glu237, can be found on the protein surface. We propose that the conformational change is likely due to the ligand (FAD) binding, rather than the hydrophobic reagent (dioxane) and low pH, by following reasons. Crystals of the as-purified MTHFR could be obtained without the hydrophobic reagent (dioxane). Determined models form crystals with or without dioxane are the same, indicating that the reagent should not be related to the conformational change. On one hand, Arg197 forming salt bridge with Glu193 in the FAD-replete (homodimer) MTHFR, on the other hand, a salt bridge between Arg197 and Glu267 could be formed in the as-purified MTHFR. Given the low pH (at pH∼4.4, we used to obtain crystals of the as-purified MTHFR) could disrupt the salt bridge of Arg197-Glu193, the same amino acid residue would not form salt bridge with other Glu residues. Both Glu193 and Glu 267 are on protein surface without any specific interaction to other residues. This suggests that the structure [file pone.0023716.s002.tif]

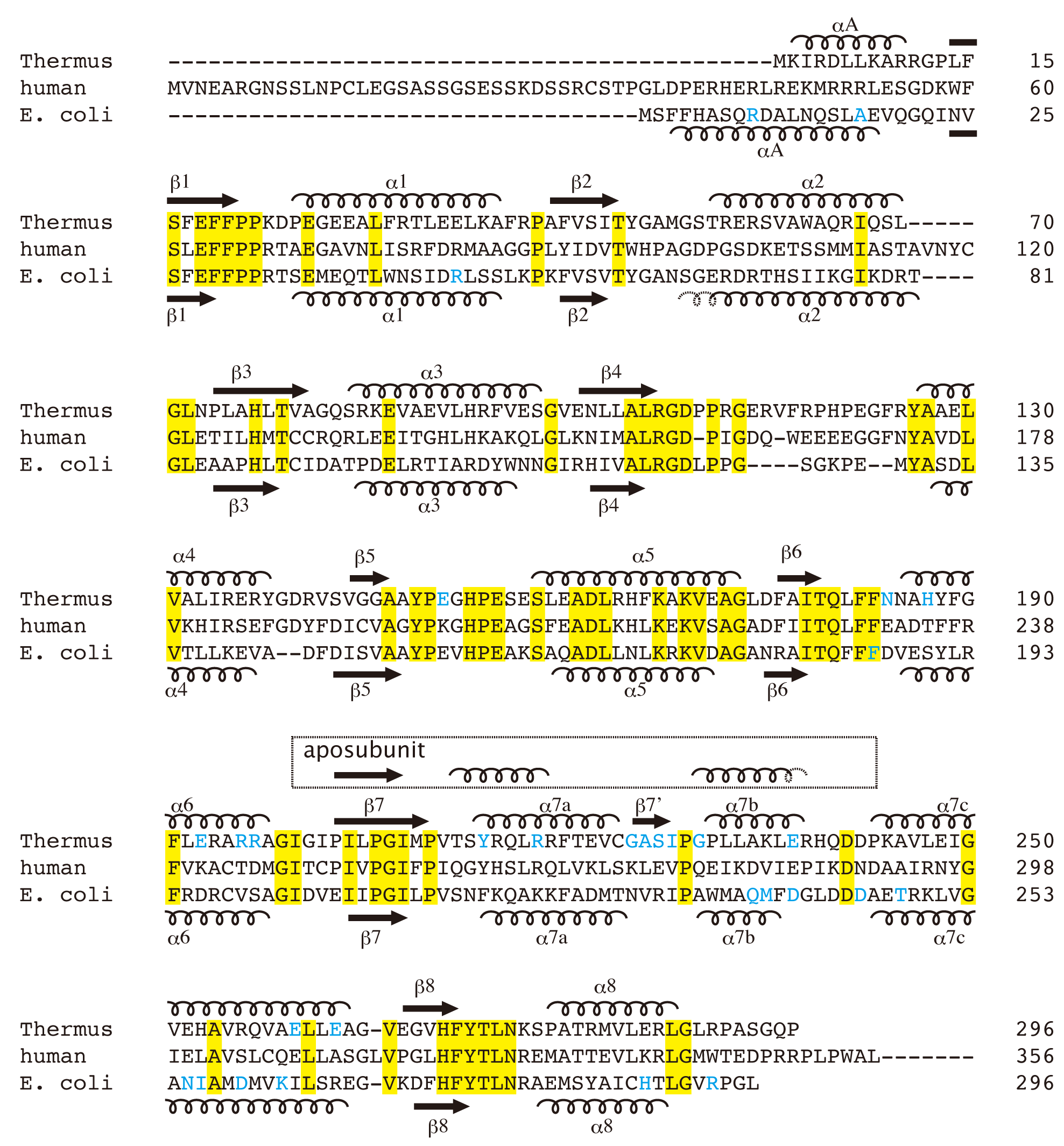

Supplement: Figure S3 — Alignment of the deduced amino acid of MTHFRs. Alignment of the deduced amino acid of MTHFRs from T. thermophilus HB8, E. coli K12, and human (the catalytic domain) is shown. The secondary structure of both bacterial MTHFRs is illustrated in this figure. Assignment of secondary structure of E. coli MTHFR was cited from Pejchal et. al. (Biochemistry (2005) 44, 11447-11457). Conserved amino acids between these three species are highlighted by yellow. For Thermus MTHFR, amino acid residues involved in the intermolecular interface of either hetero- or homo-dimer are colored in cyan. In E. coli MTHFR, the amino acid residues found at the interface are also shown in cyan. Amino acid residues forming the intermolecular interface were not conserved between E. coli and Thermus MTHFR. (TIF) [file pone.0023716.s003.tif]
